# Metformin ameliorates BSCB disruption by inhibiting neutrophil infiltration and MMP‐9 expression but not direct TJ proteins expression regulation

**DOI:** 10.1111/jcmm.13235

**Published:** 2017-07-12

**Authors:** Di Zhang, Qian Tang, Gang Zheng, Chenggui Wang, Yifei Zhou, Yaosen Wu, Jun Xuan, Naifeng Tian, Xiangyang Wang, Yan Wu, Huazi Xu, Xiaolei Zhang

**Affiliations:** ^1^ Department of Orthopaedics The Second Affiliated Hospital and Yuying Children's Hospital of Wenzhou Medical University Wenzhou Zhejiang Province China; ^2^ Zhejiang Provincial Key Laboratory of Orthopaedics Wenzhou Zhejiang Province China; ^3^ Department of Orthopaedics The Second Affiliated Hospital School of Medicine Zhejiang University Hangzhou Zhejiang Province China; ^4^ Chinese Orthopaedic Regenerative Medicine Society Wenzhou Zhejiang Province China

**Keywords:** blood‐spinal cord barrier, metformin, neutrophil, matrix metalloproteinases, inflammation, spinal cord injury

## Abstract

Blood‐spinal cord barrier (BSCB) disruption is a major process for the secondary injury of spinal cord injury (SCI) and is considered to be a therapeutic target for SCI. Previously, we demonstrated that metformin could improve functional recovery after SCI; however, the effect of metformin on BSCB is still unknown. In this study, we found that metformin could prevent the loss of tight junction (TJ) proteins at day 3 after SCI *in vivo*, but *in vitro* there was no significant difference of these proteins between control and metformin treatment in endothelial cells. This indicated that metformin‐induced BSCB protection might not be mediated by up‐regulating TJ proteins directly, but by inhibiting TJ proteins degradation. Thus, we investigated the role of metformin on MMP‐9 and neutrophils infiltration. Neutrophils infiltration is the major source of the enhanced MMP‐9 in SCI. Our results showed that metformin decreased MMP‐9 production and blocked neutrophils infiltration at day 1 after injury, which might be related to ICAM‐1 down‐regulation. Also, our *in vitro* study showed that metformin inhibited TNF‐α‐induced MMP‐9 up‐regulation in neutrophils, which might be mediated *via* an AMPK‐dependent pathway. Together, it illustrated that metformin prevented the breakdown of BSCB by inhibiting neutrophils infiltration and MMP‐9 production, but not by up‐regulating TJ proteins expression. Our study may help to better understand the working mechanism of metformin on SCI.

## Introduction

Blood‐spinal cord barrier is an essential component for the homeostasis of spinal cord. The barrier function of BSCB is based on the non‐fenestrated endothelial cells and their accessory structures, including the basement membrane, pericytes and astrocytic end feet processes, which form a tight seal owing to the presence of well‐developed tight junction (TJ) blocking the entry of plasma components and blood cells into the spinal cord 1, 2. The BSCB function is essential and particularly important in the precise control of the specialized microenvironment 1, 3, 4. When BSCB is destroyed by various disorders including traumatic spinal cord injury (SCI); the inflammatory cells and plasma components would enter the area of injury, and neurotoxic products are generated to compromise neuronal and synaptic functions 5, 6. These changes above finally will aggravate the secondary injury of SCI and lead to permanent neurological deficits. Thus, drugs preventing BSCB disruption might attenuate nervous system symptoms and improve the prognosis of SCI.

Matrix Metalloproteinases (MMPs) are calcium‐requiring, zinc‐containing endopeptidases, which participate mainly in the degradation of the extracellular matrix. They have been considered to play important roles in development, extracellular matrix remodelling, wound healing, angiogenesis and neurogenesis 7, 8, 9. They also have been reported in various central nervous system (CNS) injury including traumatic SCI, in which MMPs activation is responsible for the breakdown of BSCB 10. During the acute SCI, infiltrating neutrophils and other types of cell secrete MMPs, which further compromise the integrity of BSCB and result in neuro‐inflammation and cell death 7, 11. Among MMPs, the MMP‐9 plays a critical role in BSCB disruption and inflammation after acute SCI, suppressing MMP‐9 activity inhibits BSCB permeability and improve functional recovery 10, 12, 13. In MMP‐9 knockout mice, a significant reduction of motor deficits after traumatic brain injury is observed compared to wild mice 14. Therefore, we suggested that limiting the level of MMP‐9 would prevent the BSCB disruption after SCI.

Emerging evidence indicated that infiltrated neutrophils are the major source of the MMP‐9 in SCI 15, 16, 17. As the first inflammatory cells, neutrophils infiltrate the site of injury following traumatic CNS injury, and it peaks at 24 hrs after injury 18. The infiltration of neutrophils is regulated by intercellular adhesion molecule‐1 (ICAM‐1), which expresses on endothelial cells, and high expression of ICAM‐1 facilitates neutrophil adhesion and tissue infiltration 18. The infiltrated neutrophils accumulates in the inflammatory core of a tissue lesion to secret MMPs, which are oxidative and tissue‐degrading enzymes 19. It is reported that neutrophil‐derived factors could affect neuronal survival, and cell–cell contact between neutrophils and neurons could cause neurons insult 20, 21. Blocking neutrophils infiltration is found to promote SCI recovery 10, 22.

Metformin is one of the most commonly prescribed hypoglycaemic agents for the therapy of type 2 diabetes mellitus. Aside from the effect on ameliorating hyperglycaemia, metformin exerts therapeutic effects on various CNS disorders, including Parkinson's disease, Huntington's disease and ischaemic brain injury 23, 24, 25, 26. Our previous study has shown that metformin could improve functional recovery after SCI 27. In addition, it is found that metformin inhibits the invasion of tumour cells by decreasing MMP‐9 expression 28, 29. Recently, it also is reported that blood‐brain barrier (BBB) disruption could be attenuated by metformin administration after cerebral ischaemia 23. However, whether metformin treatment could prevent BSCB disruption to exert neuroprotective effects on acute SCI and underlying mechanisms remain to be elucidated.

In the present study, we investigated the effects of metformin on BSCB *in vivo* and *in vitro*. We found that metformin could prevent the loss of TJ proteins including Occludin, Claudin‐5, and ZO‐1 *in vivo*, but not *in vitro* in endothelial cells under normal condition or oxygen‐glucose deprivation (OGD) condition. This indicated that the therapeutic effect of metformin on BSCB disruption might not be mediated *via* up‐regulating TJ proteins directly, but *via* inhibiting TJ proteins degradation. Furthermore, we demonstrated that metformin could decrease MMP‐9 production and block neutrophils infiltration which might be related to ICAM‐1 down‐regulation. Also, we found that metformin inhibited MMP‐9 up‐regulation in neutrophils by an AMPK‐dependent pathway. Together, it illustrated that metformin prevented the breakdown of BSCB by inhibiting neutrophils infiltration and MMP‐9 production. Our study may help to better understand the working mechanism of the therapeutic effect of metformin on SCI.

## Materials and methods

### Reagents and antibodies

Metformin hydrochloride was purchased from MedChemExpress (Princeton, NJ, USA). The RPMI 1640 medium and foetal bovine serum (FBS) were purchased from Invitrogen (Carlsbad, CA, USA). Anti‐p‐AMPK, anti‐AMPK, goat anti‐rabbit and antimouse IgGHRP were purchased from Cell Signaling Technology (Danvers, MA, USA). Anti‐β‐actin, anti‐ZO‐1, anti‐Occludin, anti‐Claudin‐5, anti‐ICAM‐1 were purchased from Santa Cruz Biotechnology (Santa Cruz, CA, USA). Anti‐ly6G, anti‐CD31, anti‐MMP‐2, anti‐MMP‐9 were obtained from Abcam (Cambridge, CA, USA). An enhanced chemiluminescence (ECL) kit was purchased from Bio‐Rad (Hercules, CA, USA). N,N′‐dimethylformamide was obtained from JinSan (Wenzhou, China). AMPK inhibitor Compound C, Evans blue and all of the other reagents were purchased from Sigma‐Aldrich (St. Louis, MO, USA) unless otherwise specified.

### Experimental design

Adult female Sprague–Dawley rats (220–250 g) were purchased from the Animal Center of the Chinese Academy of Sciences in Shanghai, China. The protocol for animal care and use conformed to the Guide for the Care and Use of Laboratory Animals from the National Institutes of Health and was approved by the Animal Care and Use Committee of Wenzhou Medical University. All animals were housed in standard temperature conditions with a 12‐hr light/dark cycle and regularly fed with food and water. SCI rats were performed as described previously 30. Following sodium pentobarbital (65 mg/kg intraperitoneal injection) anaesthesia, rats were positioned on a cork platform. The skin was incised along the midline of the dorsum to expose the vertebral column and to perform a laminectomy carried out at the T9 level. The exposed spinal cord was subjected to crushing injury by compression with a vascular clip (30 g force; Oscar, China) for 1 min. Sham group rats received the same surgical procedure but sustained no impact injury, though the spinal cord was left exposed for 1 min. Metformin was diluted with normal saline, to achieve a final metformin concentration of 20 mg/ml. Following the spinal cord injury, the metformin solution was injected intraperitoneally to deliver a dose of 50 mg/kg/day until the rats were killed. Equivalent normal saline injections were administered for vehicle control. Following treatment with metformin, animals were treated uniformly until the final analysis of the data. All animals showed no significant side effects resulting from drug treatment such as mortality or signs of infectious disease during these experiments.

### Human brain microvascular endothelial cells culture and treatment

Human brain microvascular endothelial cells (HBMECs) were purchased from ScienCell Research Laboratories (ScienCell Research Laboratories, San Diego, CA, USA). Cells were cultured in endothelial cell medium (ScienCell Research Laboratories) and incubated in a humidified atmosphere contain 5% CO_2_ at 37°C. Cells were subcultured into 35‐mm dishes and treated with metformin (1 mM) for 24 and 48 hrs in conventional cell incubator, and then the cells were tested by Western blot. To mimic SCI condition, the cells were subcultured into 35‐mm dishes, and confluent endothelial cells were exposed to a hypoxia chamber for 12 hrs after overnight starvation with 0.5% foetal calf serum. Before OGD, the medium was then changed to sugar‐free basic medium with metformin (1 mM). After OGD treatment, cells were tested by Western blot.

### Primary neutrophil culture and treatment

To obtain rat neutrophils, the blood from adult (220–250 g) Sprague–Dawley rats was collected and laminated on the Percoll gradient with layer densities of 45%, 54% and 63%. After a gradient centrifugation of the blood (550 g for 15 min.), neutrophils were detected between the second and third layers. The cells were collected and additionally purified by erythrocyte lysis with ACK buffer. The granulocytes were then washed with HBSS buffer and diluted in RPMI media with 10% (v/v) heat‐inactivated FBS. The neutrophils were plated in the 6‐well dish with a density of 100,000 cells/cm^2^ 31. Neutrophils were treated with TNF‐α (100 ng/ml), metformin (1 mM) and compound C (50 μM) for 6 hrs in conventional cell incubator.

### Locomotion test

The Basso, Beattie and Bresnahan (BBB) scores were assessed by two independent examiners who were blinded to treatment to score locomotion recovery in an open field scale at 1, 3, 7 and 14 days post‐operation 30, 32. Briefly, the BBB locomotion rating scale scores range from 0 points (complete paralysis) to 21 points (normal locomotion). The scale was developed using the natural progression of locomotion recovery in rats with thoracic SCI.

### Evans blue dye assays

At 3 days after SCI, rats were injected 2% Evans blue dye (2 ml/kg, i.v). Two hours after injection, rats were anaesthetized with sodium pentobarbital (65 mg/kg, i.p), then perfused with 0.9% normal saline. The injured spinal cord tissues of EB were weighed and immersed in N,N′‐dimethylformamide at 50°C for 72 hrs. The optical density of the supernatant was examined with enzyme‐labelled metre (at an excitation wavelength of 620 nm and an emission wavelength of 680 nm). Dye in samples was determined as μg/g of tissue from a standard curve plotted using known amounts of dye. Rats were fixed by perfusion with 4% paraformaldehyde at 2 hr after EB injection. The spinal cord tissues were cut into 20‐μm thickness at −20°C using frozen section machine, then the sections were observed.

### Oedema Measurement

Animals were assessed for oedema at 3 days after SCI using the wet weight/dry weight method as described previously 33. Briefly, animals were administered a lethal injection of pentobarbital, and the spinal cord was removed rapidly. The spinal cord was cut into 10 mm segments, and the wet weight was obtained. Spinal cord segments were then oven dried at 100°C for 48 hrs before the dry weight was measured. The percentage of water content was calculated as: (wet weight − dry weight)/wet weight × 100.

### Haematoxylin‐Eosin (HE) staining

Sham and SCI rats (*n* = 5) were deeply re‐anaesthetized with sodium pentobarbital (65 mg/kg i.p) and perfused with 0.9% NaCl, followed by 4% paraformaldehyde in 0.01 M phosphate‐buffered saline (PBS, pH = 7.4) at 7 days after surgery. Tissue segments containing the lesion (1 cm on each side of the lesion) were paraffin embedded. Transverse paraffin sections (5 mm thick) were mounted on poly‐L‐lysine‐coated slides for histopathological examination by HE staining. The HE was performed is as follow: sections were re‐hydrated in 100% alcohol, 5 min. twice, 95% alcohol and 70% alcohol each for 3 min. The sections were then washed in water, stained in Harris haematoxylin solution for 5 min. After that, the sections were washed in running tap water for 8 min. and differentiated in 1% acid alcohol for 30 sec., blued in 0.2% ammonia water for 30 sec. The samples were washed in running tap water for 5 min. Next, Eosin–Phloxine solution used to stain sections for 1 min. Dehydrated through 95% alcohol, 100% alcohol, each for 5 min. and cleared in two changes of xylene for 2 × 5 min. Finally, the sections were mounted with mounting medium. All images were captured on a Nikon ECLIPSE Ti microscope (Nikon, Japan).

### Gelatin zymography

Gelatin zymography was performed as described previously 12. Briefly, the epicentre of injured spinal cord (0.5 cm in length) was homogenized in lysis buffer containing 50 mM Tris‐HCl, pH 8.0, 150 mM NaCl, 1% NP‐40, 0.5% deoxycholate and 0.1% SDS. After determination of protein concentration of the homogenates, 40 μg of protein were loaded onto 10% SDS‐polyacrylamide gels, copolymerized with gelatin (1 mg/ml, Sigma‐Aldrich). After electrophoresis, renaturation was achieved by incubation of the gel in 2.5% Triton X‐100 for 30 min. and in substrate buffer (50 mM Tris‐HCl, pH 8.5, 5 mM CaCl_2_) for 48 hrs at 37°C. The gel was stained with Coomassie blue solution for 4 hr and then de‐stained with 40% methanol/10% acetic acid. For quantitative analysis, gels were scanned, and the positive band was measured using NIH Image J software (NIH). Data were expressed as the values related to uninjured samples.

### Fluorimetric assay for matrix metalloprotease‐9 activity

MMP‐9 activity was measured using the SensoLyte 520 MMP‐9 Assay Kit according to the manufacturer's protocol (AnaSpec) as previous report 10. Briefly, tissues were homogenized with the assay buffer containing 0.1% Triton X‐100, and centrifuged for 15 min. at 10,000 *g* at 4°C. The supernatant was collected. And the supernatants were incubated with 4‐aminophenylmercuric acetate for 1 hr at 37°C. Then, MMP‐containing samples were added to a 96‐well plate (50 μl/well), and MMP‐9 substrate (50 μl/well) was added to the sample and control wells. After shaking the plate gently for 30 sec., the reagents were incubated at 37°C for 1 hr. Then, 50 μl of stop solution was added and mixed, and the fluorescence intensity was measured at an excitation wavelength of 490 nm and an emission wavelength of 520 nm.

### Real‐time polymerase chain reaction

Total RNA (0.5 μg) was extracted from the tissue using TRIzol reagent (Invitrogen) according to manufacturer's protocol. First‐strand cDNA was synthesised by a reverse transcriptase kit (Promega, Madison, USA) according to the manufacturer's instructions. Real‐time amplification, using SYBR Green Supermix (QPK‐212, Tokyo, Japan) and a Light Cycler480 system (Roche, Indianapolis, USA), was performed using the following sequences: MMP‐9 forward: 5′‐AACCCTGGTCACCGGACTTC‐3′, reverse: 5′‐CACCCGGTTGTGGAAACTCAC‐3′; β‐actin forward: 5′‐AAGATCCTGACCGAGCGTGGC‐3′, reverse: 5′‐CAGCACTGTGTT GGCATAGAGG‐3′. The q‐PCR conditions were as follows: 2 min. at 50°C and 10 min. at 95°C, followed by a total of 40 cycles of 2 temperature cycles (15 sec. at 95°C and 1 min. at 60°C). β‐actin was used as an internal control. The relative expression levels were analysed using the 2^−ΔΔCt^ method with the relative expression software tool 34.

### Myeloperoxidase activity

Myeloperoxidase (MPO) activity, an indicator of polymorphonuclear leucocyte accumulation, was determined as previously described 35. At 1 day following SCI, spinal cord tissues were obtained and weighed, and each piece was homogenized in a solution containing 0.5% (w/v) hexadecyltrimethyl‐ammonium bromide dissolved in 10 mM potassium phosphate buffer (pH = 7) and centrifuged for 30 min. at 20,000 *g* at 4°C. The supernatant was then reacted with a solution of tetramethylbenzidine (1.6 mM) and 0.1 mM hydrogen peroxide. The rate of change in absorbance was measured spectrophotometrically at 650 nm. MPO activity was defined as the quantity of enzyme degrading 1 μmol peroxide/min. at 37°C and was expressed in milliunits/g of wet tissue.

### Enzyme‐linked immunosorbent assay

The rats were killed at 1 day after SCI, neutrophils were treated with TNF‐α (100 ng/ml) alone, or TNF‐α and metformin (1 mM) for 6 hr. Cell culture media were collected. Protein concentrations were measured with commercial ELISA quantification kits for MMP9 (R&D Systems) according to the manufacturer's instructions.

### Western blot analysis

Spinal cord tissue samples were removed at designated time after surgery, and the spinal cords from the T7 to T10 levels around the lesion epicentre were excised, a spinal cord segment (0.5 cm length) at the contusion epicentre was dissected and rapidly stored at −80°C for Western blotting. Briefly, frozen animal spinal cord tissues and cells were homogenized in ice‐cold lysis buffer containing 50 mM Tris‐HCl pH 8.0, 150 mM NaCl, 1% NP‐40, 0.5% deoxycholate, 0.1% SDS, 10 mM Na2P2O7, 10 mM NaF, 1 mg/ml aprotinin, 10 mg/ml leupeptin, 1 mM sodium vanadate and 1 mM PMSF. After homogenizing, tissues were centrifuged at 11,792 g, for 15 min. at 4°C. The equivalent of 60 μg of total protein was loaded onto SDS‐PAGE and transferred to PVDF membrane (Bio‐Rad). The membrane was blocked with 5% non‐fat milk in TBS with 0.1% Tween 20 for 90 min., and then incubated overnight at 4°C with primary antibody solutions according to the manufacturer's recommendations. Then the membranes were washed with TBS, and primary antibodies were detected with horseradish peroxidase‐conjugated secondary antibodies. Signals were visualized using the ChemiDicTM XRS + Imaging System (Bio‐Rad). Experiments were repeated three times.

### Immunofluorescence staining

Spinal cord sections were incubated with 10% normal goat serum for 1 hr at room temperature in PBS containing 0.1% Triton X‐100. They were then incubated with the appropriate primary antibodies overnight at 4°C in the same buffer. The following primary antibodies were used, based on differing targets: anti‐CD31 (1:200), anti‐Ly6G (1:100), anti‐MMP‐9 (1:100) and anti‐Occludin (1:100). After primary antibody incubation, sections were washed for 4 × 10 min. and then incubated with Alexa Fluor 488‐conjugated anti‐IgG or Texas red‐conjugated anti‐IgG secondary antibodies for 1 hr at room temperature. Sections were rinsed three times with PBS and incubated with 4, 6‐diamidino‐2‐phenylindole (DAPI) for 10 min. and finally washed in PBS and sealed with a coverslip. Cells, grown on 14*14 mm microscopic glass were washed with ice‐cold PBS, fixed with 4% paraformaldehyde for 30 min., then washed with ice‐cold PBS and blocked in 5% BSA for 1 hr. Then cells were incubated with primary antibodies at 4°C overnight. Cells were washed with PBS followed by incubation with Alexa Fluor 488‐conjugated anti‐IgG or Texas red‐conjugated anti‐IgG secondary antibodies for 1 hr at room temperature. After washing with PBS, the nuclei were stained with DAPI for 10 min. and finally washed in PBS and sealed with a coverslip. All images were captured on a Nikon ECLIPSE Ti microscope (Nikon, Japan).

### Statistical analysis

Data are presented as mean ± SEM. Statistical significance was examined using Student's *t*‐test when there were two experimental groups. For more than two groups, statistical evaluation of the data was performed with the one‐way ANOVA test, Tukey's multiple comparison is used as a post hoc analysis. For all analyses, *P* < 0.05 was considered significant.

## Results

### Metformin attenuated BSCB permeability and improved functional recovery after SCI

**Figure 1:**
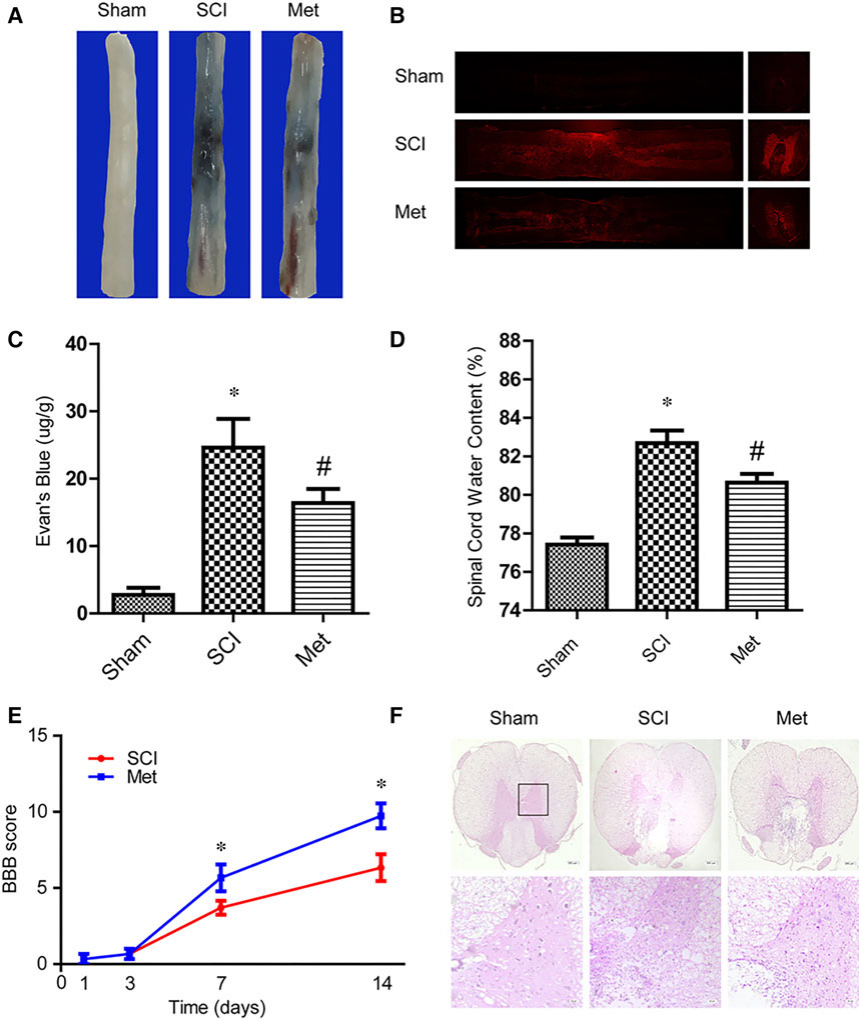
Metformin attenuated BSCB permeability and improved functional recovery after SCI. (**A**) Representative whole spinal cords showed that Evan's Blue dye permeabilized into spinal cord at 3 days after SCI, *n* = 5. (**B**) Representative fluorescent images of Evans Blue Dye extravasation at 3 days. (**C**) Quantification of the amount of Evan's Blue at 3 days (μg/g), * represents *P* < 0.05 versus the sham group, *n* = 5, # represents *P* < 0.05 versus the SCI group, *n* = 5. (**D**) Effect of metformin on the water content in the injured spinal cord areas at 3 days after SCI. * represents *P* < 0.05 versus the sham group, *n* = 5, # represents *P* < 0.05 versus the SCI group, *n* = 5. (**E**) The Basso, Beattie and Bresnahan (BBB) scores,* represents *P* < 0.05 versus the SCI group, *n* = 5. (**F**) HE staining at 7 days after injury. Scale bars are 200 and 20 μm.

To evaluate the therapeutic effect of metformin on BSCB permeability after acute traumatic SCI, BSCB function was investigated for 3 days after insult by Evan's Blue assay and water content. Compared with sham group, the amount of Evan's Blue dye extravasation had a significant increase after SCI, which indicates BSCB leakage (Fig. 1A). While metformin administration reduced the amount of Evan's Blue dye extravasation at 3 days after SCI as compared with SCI group. For injured spinal cord the fluorescence of Evan's Blue was higher than Sham group, but metformin decreased the fluorescence intensity (Fig. 1B). Qualitative analysis of Evan's Blue also showed the same results (Fig. 1C). The water content of spinal cord tissue was significantly increased after SCI, but metformin attenuated SCI‐induced oedema (Fig. 1D). All of these results indicated that metformin prevented the BSCB dysfunction. And Functional recovery was then assessed using BBB scores for movement. Metformin administration significantly enhanced the BBB scores within 7–14 days after injury, compared to vehicle‐treated controls (Fig. 1E). Histomorphology differences between the sham, SCI and metformin treatment groups were investigated by HE staining at 7 days after injury. SCI led to severe damage of the dorsal white matter and central grey matter, while metformin treatment alleviated cavity formation and preserved more tissue in the dorsal white matter and central grey matter (Fig. 1F). These results revealed a significant improvement in functional recovery and tissue preservation in metformin treated rats.

### Metformin prevented the loss of TJ proteins after SCI

**Figure 2:**
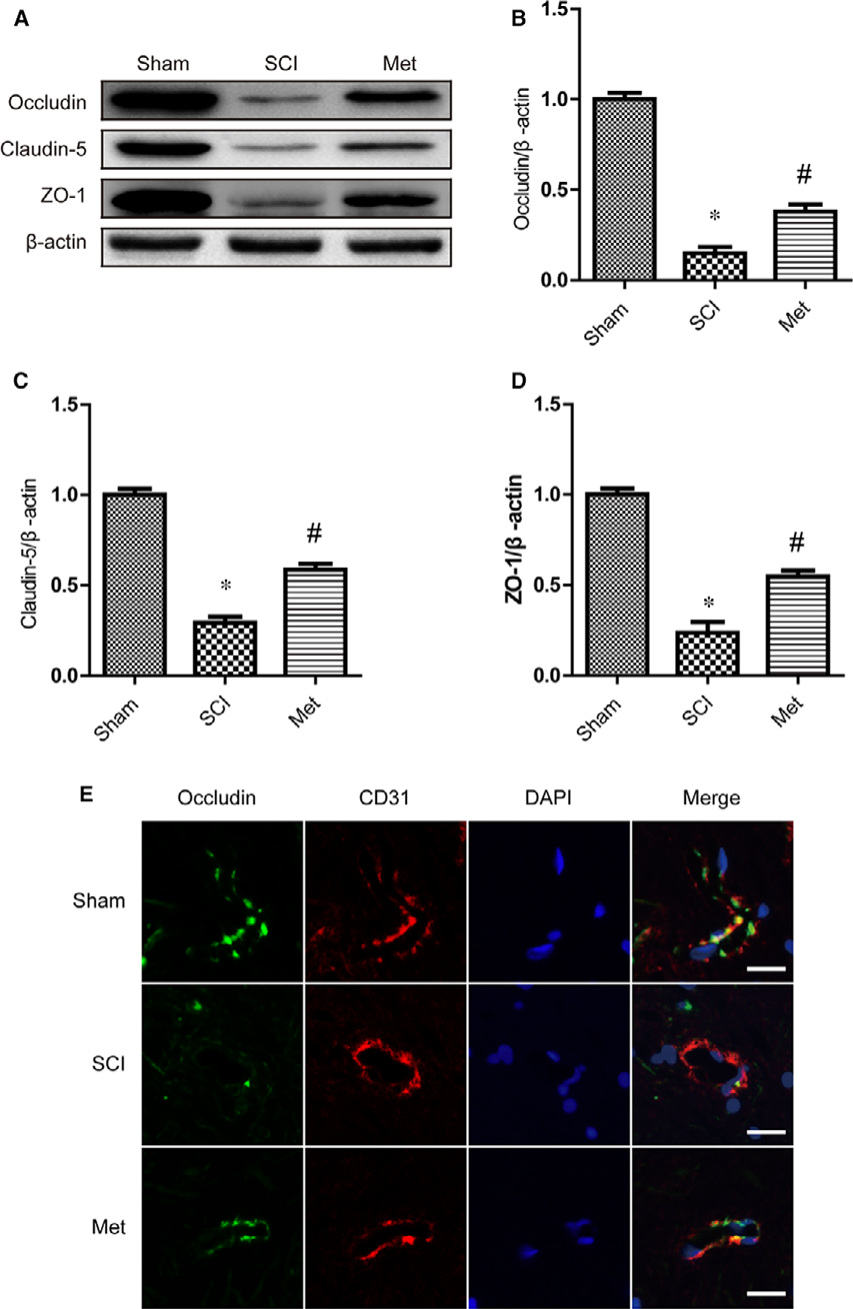
Metformin prevented the loss of TJ proteins after SCI. (**A**–**D**) Representative Western blots and quantification data of Occludin, Claudin‐5, ZO‐1 and β‐actin at 3 day after injury, * represents *P* < 0.05 versus the sham group, *n* = 5, # represents *P* < 0.05 versus the SCI group, *n* = 5. (**E**) Double staining for Occludin (green)/CD31 (red) of sections from the spinal cord in each group rats. Results are from three independent sections between 4 and 5 mm from the injury epicentre. Scale bars are 20 μm.

To investigate endothelial cells of blood vessels permeability, which involves in the integrity BSCB, we examined the alterations of SCI‐induced TJ proteins, including Occludin, Claudin‐5 and ZO‐1, and the effect of metformin on these alterations by Western blot. As the results shown, the TJ proteins were obviously decreased after SCI (Fig. 2A, B, C and D). But, metformin treated rats showed Occludin, Claudin‐5 and ZO‐1 hyper‐expression at 3 days. To reconfirmed that, we also detected TJ proteins at 5 and 7 days after injury, and the Western blot results showed the same changes at different time‐point (see Fig. S1A and B). Occludin/CD31 (a marker of endothelial cell) double immunofluorescence staining was performed to observe TJ proteins distribution *in situ* at 3 days after SCI. Metformin administration attenuated the decrease of Occludin in endothelial cells of blood vessels around the epicentre of injury site (Fig. 2E). These data indicated that metformin prevented BSCB dysfunction by up‐regulating TJ proteins after SCI.

### Metformin had no significant effect on the level of TJ proteins in endothelial cells

**Figure 3:**
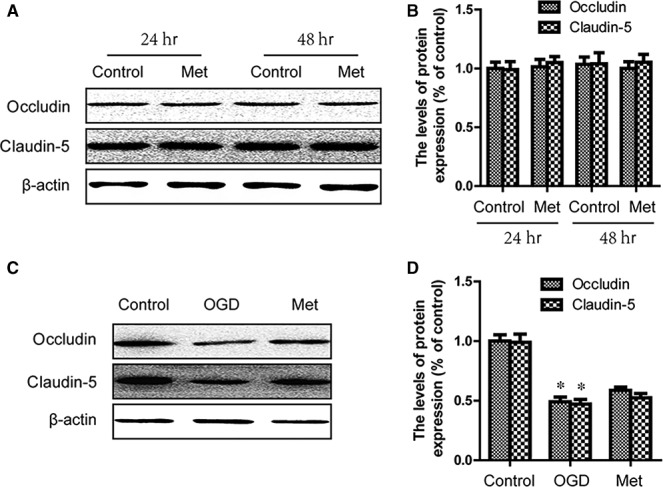
Metformin had no significant effect on the level of TJ proteins in endothelial cells. (**A**,** B**) Endothelial cells were treated for 24 and 48 hrs with metformin (1 mM). Western blots were performed using the indicated antibodies. (**C**,** D**) Endothelial cells were subjected to OGD for 12 hrs. Western blots were performed using the indicated antibodies. * represents *P* < 0.05 versus the control group, *n* = 5.

To confirm the potential mechanism of metformin‐mediated regulation TJ proteins, we treated HBMECs with metformin. Western blot analyses were performed with the HBMECs at 24 and 48 hrs after metformin treatment. As the results shown, metformin could not significantly increase the level of Claudin‐5 and Occludin in endothelial cells (Fig. 3A and B). To investigate the effect of metformin on TJ protein under pathological conditions, the endothelial cells were exposed to a hypoxia chamber to mimic SCI condition. And we found that metformin increased, but not significantly, the level of Claudin‐5 and Occludin in endothelial cells under OGD condition (Fig. 3C and D). All of these results suggested that metformin might not directly up‐regulate the level of TJ proteins, or that the effect of metformin on up‐regulation of TJ proteins might be not obvious in endothelial cells.

### Metformin inhibited MMP‐9 production after SCI

**Figure 4:**
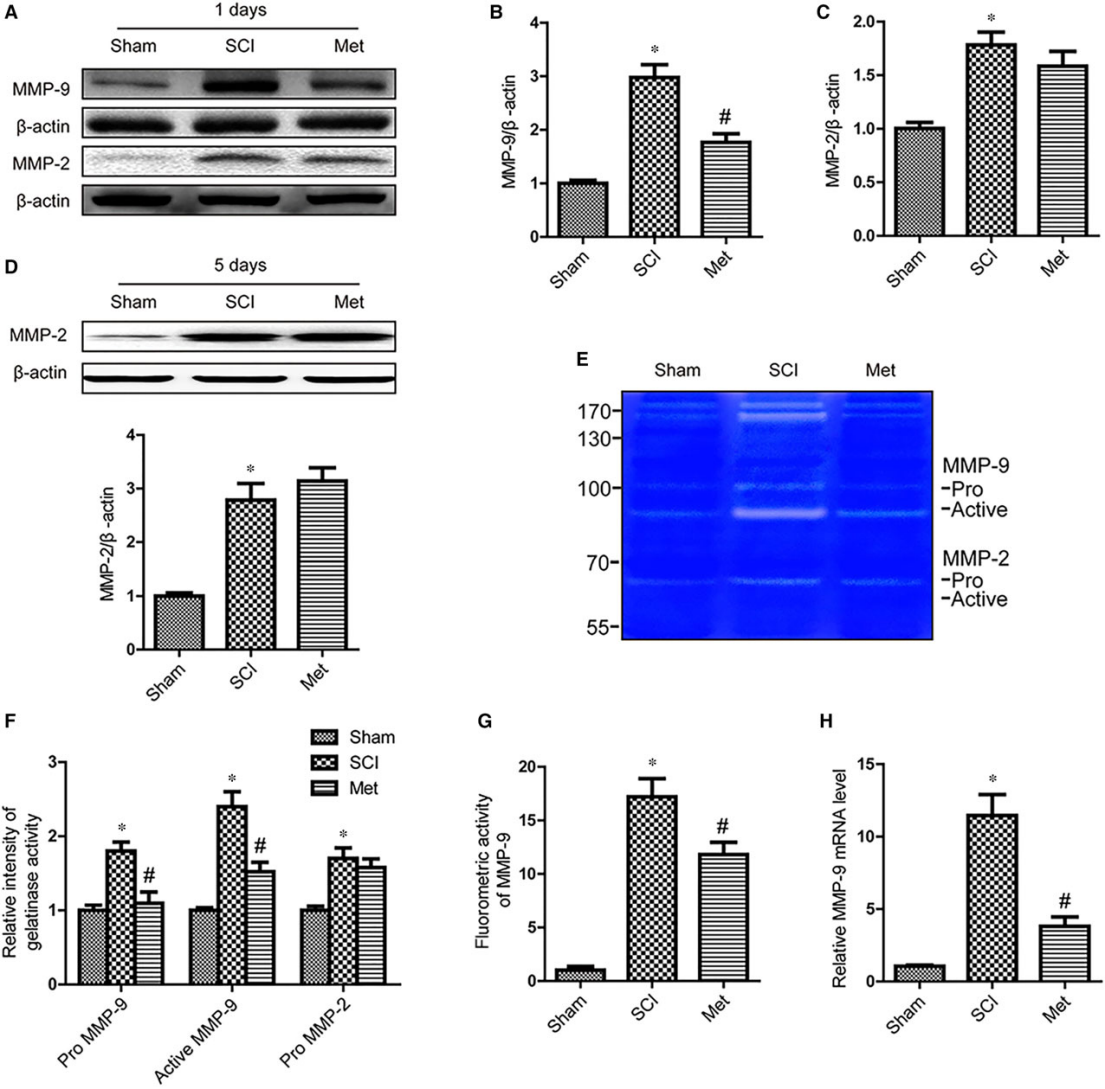
Metformin inhibited MMP‐9 production after SCI. (**A–C**) Representative Western blots and quantification data of MMP‐2, MMP‐9 and β‐actin at 1 day after injury, * represents *P* < 0.05 versus the sham group, *n* = 5, # represents *P* < 0.05 versus the SCI group, *n* = 5. (**D**) Representative Western blots and quantification data of MMP‐2 and β‐actin at 5 days after injury, * represents *P* < 0.05 versus the sham group, *n* = 5. (**E**,** F**) Representative zymography and quantification data of MMP‐2 and MMP‐9 at 1 day after injury, * represents *P* < 0.05 versus the sham group, *n* = 5, # represents *P* < 0.05 versus the SCI group, *n* = 5. (**G**) MMP‐9 activity was measured by fluorimetric assay, * represents *P* < 0.05 versus the sham group, *n* = 5, # represents *P* < 0.05 versus the SCI group, *n* = 5. (**H**) mRNA level of MMP‐9 at 1 day after injury, * represents *P* < 0.05 versus the sham group, # represents *P* < 0.05 versus the SCI group.

It is well known that excessive proteolytic activity of MMPs such as MMP‐9 and MMP‐2 results in TJ proteins degradation and BSCB disruption after SCI. Thus, we investigated the effect of metformin on the level of MMP‐2 and MMP‐9 after SCI. The results of Western blot indicated that up‐regulation of MMP‐9 after SCI was significantly inhibited by metformin administration (Fig. 4A and B). But the MMP‐2 revealed no significant differences (Fig. 4A and C). Because it has been reported that MMP‐2 activity maximized at 5 days after SCI, we also detected the effect of metformin on the level of MMP‐2 at 5 days after injury. And the results show that MMP‐2 could not be decreased by metformin treatment (Fig. 4D). Next, the activities of MMP‐2/MMP‐9 were analysed by gelatin zymography. We found that Pro MMP‐9, active MMP‐9 and Pro MMP‐2 activities were increased at 1 day after insult (Fig. 4E and F). While metformin treatment decreased grey value of Pro MMP‐9 and active MMP‐9 bands, which indicates weakened MMP‐9 activity. However, metformin treatment showed no significant effect on Pro MMP‐2 activity in gelatin zymography. In addition, we reconfirmed the effect of metformin on MMP‐9 activity using the fluorimetric enzyme activity assay kit. These results indicated that metformin administration markedly suppressed MMP‐9 activity at 1 day after injury as compared to SCI controls (Fig. 4G). Finally, we examined the expression of MMP‐9 mRNA at 1 day after injury by real‐time polymerase chain reaction. As shown in Figure 4H, the mRNA levels of MMP‐9 markedly increased at 1 day after injury. In addition, metformin administration significantly inhibited MMP‐9 mRNA expression at 1 day after surgery compared with the SCI controls. All of these results demonstrated that metformin treatment inhibited MMP‐9 after SCI.

### Metformin blocked neutrophils infiltration and activated AMPK after SCI

**Figure 5:**
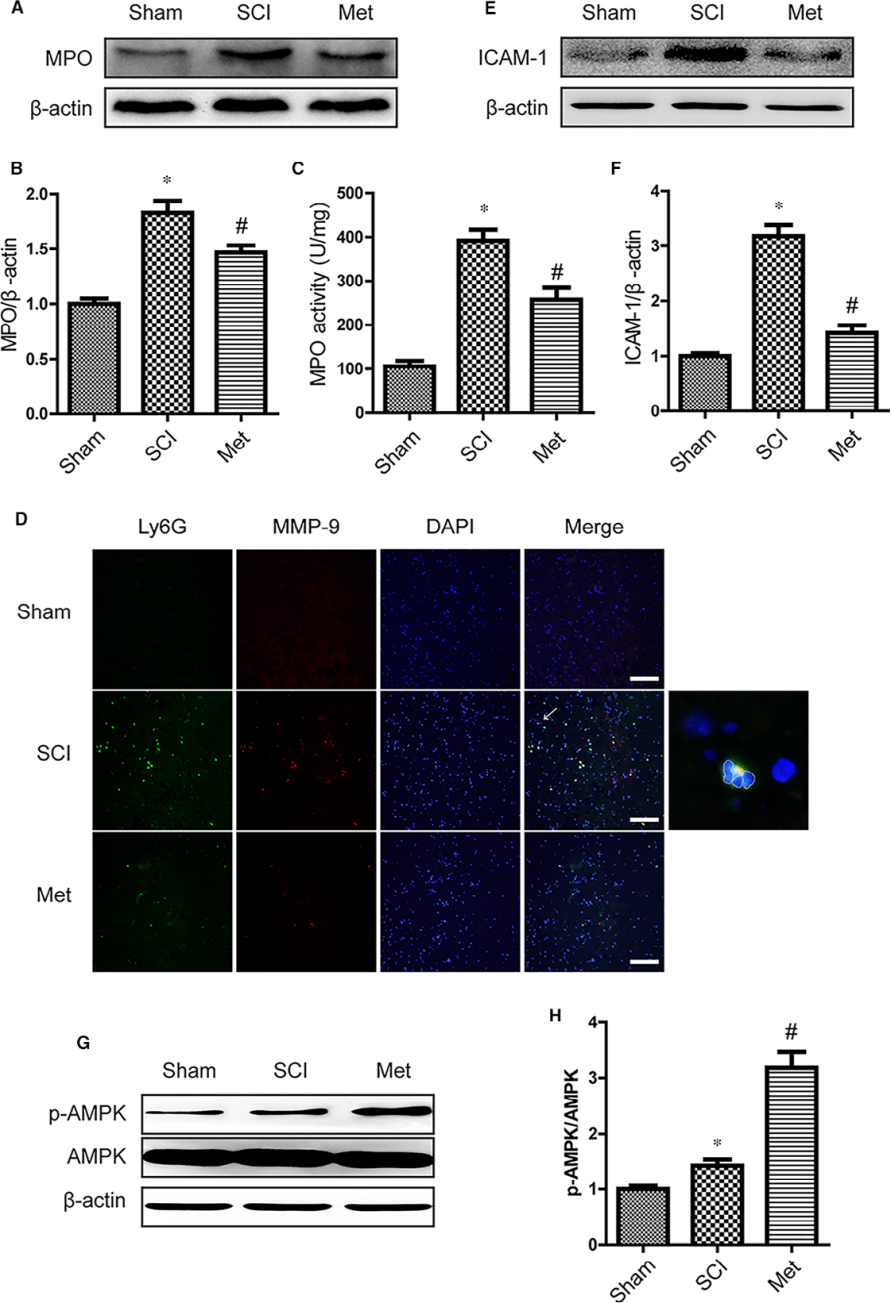
Metformin blocked neutrophils infiltration and Activated AMPK after SCI. (**A**,** B**) Representative Western blots and quantification data of MPO and β‐actin at 1 day after SCI, * represents *P* < 0.05 versus the sham group, *n* = 5, # represents *P* < 0.05 versus the SCI group, *n* = 5. (**C**) MPO activity in spinal cord from each group rats, * represents *P* < 0.05 versus the sham group, *n* = 5, # represents *P* < 0.05 versus the SCI group, *n* = 5. (**D**) Double staining for Ly6G (green)/MMP‐9 (red) of sections from the injured spinal cord in each group rats. Results are from three independent sections between 4 and 5 mm from the injury epicentre. Scale bars are 100 μm. (**E**,** F**) Representative Western blots and quantification data of ICAM‐1 and β‐actin in each group rats, * represents *P* < 0.05 versus the sham group, *n* = 5, # represents *P* < 0.05 versus the SCI group, *n* = 5. (**G**,** H**) Representative Western blots and quantification data of AMPK, p‐AMPK and β‐actin at 1 day after injury, * represents *P* < 0.05 versus the sham group, *n* = 5.

Neutrophils infiltrate lesioned tissue and play active roles in the secondary tissue damage after SCI 36. To detect the impact of metformin on neutrophil infiltration, we examined the level of myeloperoxidase (MPO) at 1 day after SCI by Western blot. Based on the analysis of the band density, we found a significant increase in the level of MPO in SCI group compared with sham controls (Fig. 5A and B). While metformin administration down‐regulated the level of MPO, which indicates a decrease in neutrophil infiltration. MPO activity test was also performed to detect neutrophilic exudate, and we observed the same result with Western blot (Fig. 5C). It is becoming more widely accepted that neutrophils contribute to the MMP‐9 production after SCI. Therefore, we assessed the level of MMP‐9 by double staining for MMP‐9 (red)/Ly6G (green, a marker of neutrophils). In contrast to sham animals only having few MMP‐9 positive (MMP‐9^+^) neutrophils, vehicle animals showed strong increase in the number of neutrophils and MMP‐9^+^ neutrophils in areas around the injury at 1 day (Fig. 5D). However, this trend was reversed by metformin administration. Because ICAM‐1 expresses in endothelial cells, which facilitates neutrophil adhesion and tissue infiltration, we analysed ICAM‐1 expression by Western blot. As the results shown, ICAM‐1 was elevated hugely after SCI (Fig. 5E and F). However, ICAM‐1 was decreased after metformin administration compared to vehicle controls. Taken together, these results indicated that metformin prevented the neutrophil infiltration and MMP‐9 activation. In addition, we detected the activation of AMPK, and we found that metformin treatment could significant activate AMPK signalling pathway after SCI, indicating that AMPK might be involved in the effect of metformin (Fig. 5G and H).

### Metformin inhibited MMP‐9 production in neutrophils *in vitro*

**Figure 6:**
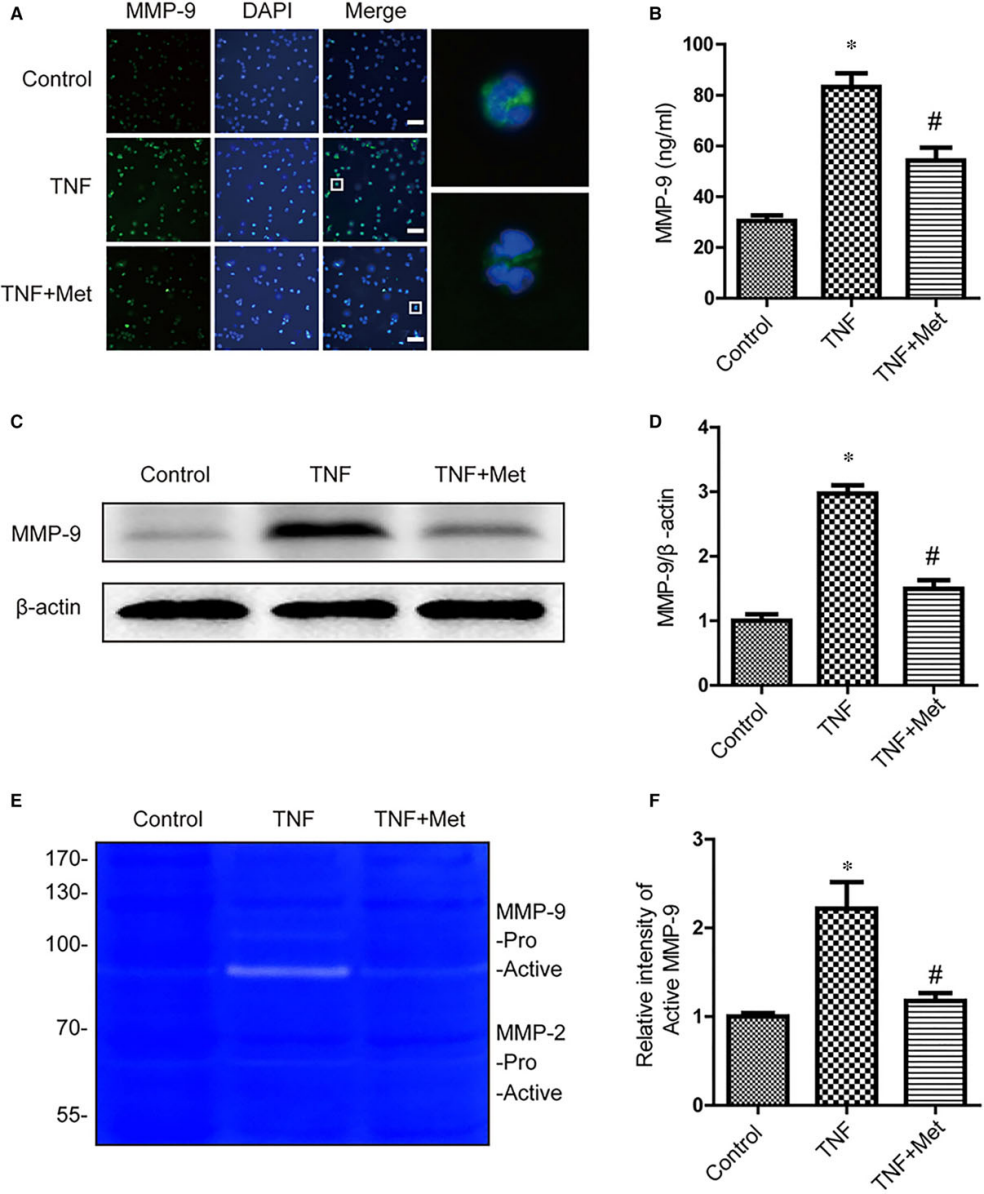
Metformin inhibited activation of MMP‐9 in neutrophils *in vitro*. Neutrophils were treated with TNF‐α (100 ng/ml) and metformin (1 mM) for 6 hr. (**A**) Representative fluorescent images of MMP‐9 in neutrophils. Scale bars are 25 μm. (**B**) The release of MMP‐9 in the culture medium was measured by ELISA assay, * represents *P* < 0.05 versus the control group, *n* = 5, # represents *P* < 0.05 versus the TNF group, *n* = 5. (**C**,** D**) Representative Western blots and quantification data of MMP‐9 and β‐actin in each group, * represents *P* < 0.05 versus the control group, *n* = 5, # represents *P* < 0.05 versus the TNF group, *n* = 5. (**E**,** F**) Representative zymography and quantification data of MMP‐9 and MMP‐2, * represents *P* < 0.05 versus the control group, *n* = 5, # represents *P* < 0.05 versus the TNF group, *n* = 5.

To confirm the effect of metformin on inhibiting MMP‐9 activation in neutrophils, we isolated neutrophils from whole blood of rat and treated with TNF‐α to induce high expression of MMP‐9 *in vitro*. Our immunostaining demonstrated that TNF‐α strongly increased the number of MMP‐9^+^ neutrophils (Fig. 6A). But we observed lesser MMP9^+^ neutrophils in the metformin and TNF‐α co‐treatment. Furthermore, the ELISA assay demonstrated a dramatic increase in MMP‐9 secretion in cell‐free supernatants after TNF‐α treatment (Fig. 6B). While, this trend was inhibited by metformin treatment. We also conducted Western blot to detect the MMP‐9 expression in neutrophils. The same results as above were observed in metformin and TNF‐α co‐treatment (Fig. 6E and F). In gelatin zymography test, TNF‐α treatment only enhanced Active MMP‐9, and metformin reversed this change. All of these results suggested that metformin suppressed activation of MMP‐9 in neutrophils.

### AMPK was involved in metformin‐mediated MMP‐9 inhibition

**Figure 7:**
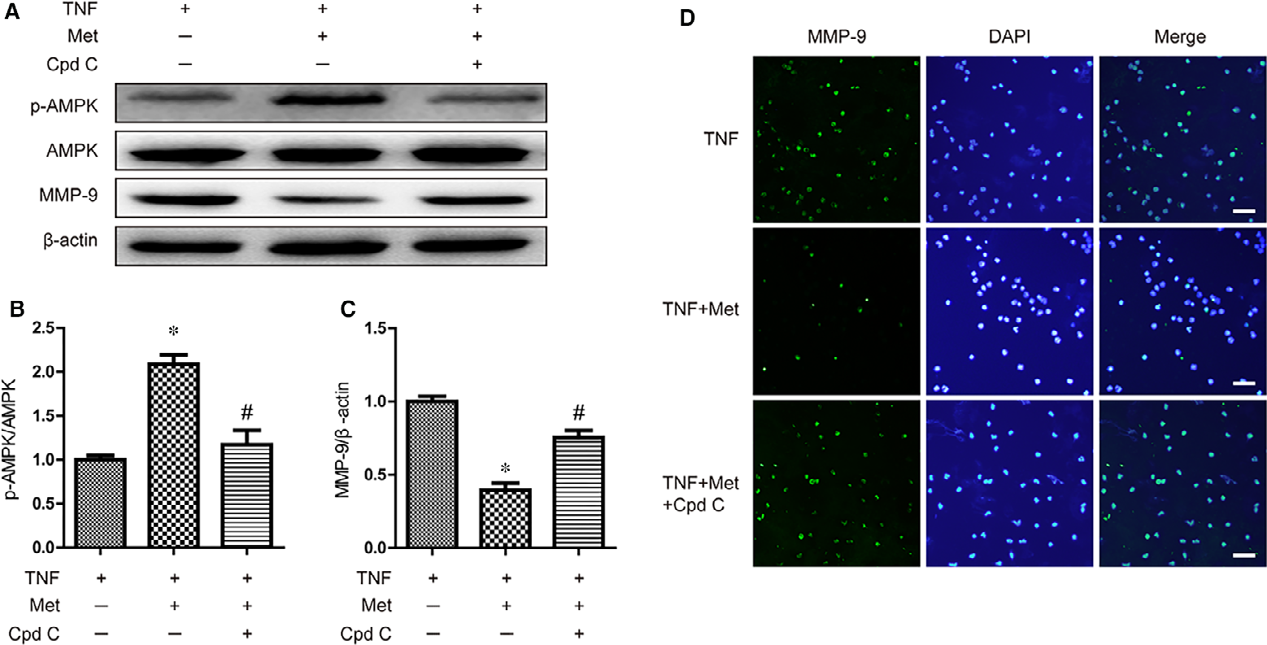
AMPK was involved in metformin‐mediated MMP‐9 inhibition in neutrophils *in vitro*. Neutrophils were treated with TNF‐α (100 ng/ml), metformin (1 mM) and compound C (50 μM) for 6 hr. (**A**–**C**) Representative Western blots and quantification data of p‐AMPK, AMPK, MMP‐9 and β‐actin in each group, * represents *P* < 0.05 versus the control group, *n* = 5, # represents *P* < 0.05 versus the TNF group, *n* = 5. (**D**) Representative fluorescent images of MMP‐9 in neutrophils. Scale bars are 25 μm.

To detect whether the effect of metformin on inhibiting MMP‐9 activation in neutrophils was AMPK dependent, Compound C (Cpd C), an classic AMPK inhibitor, was utilized to treat neutrophils 37, 38. Our immunoblot results demonstrated that the phosphorylation of AMPK was increased in metformin treatment, and Compound C significantly suppressed phosphorylation of AMPK in neutrophils co‐treatment with metformin and Compound C (Fig. 7A, B and C). Furthermore, along with the AMPK inactivation, Compound C abolished the function of metformin on inhibiting MMP‐9. And the results of immunofluorescence showed similar results (Fig. 7D). These results indicated that AMPK was involved in inhibition of MMP‐9 mediated by metformin.

## Discussion

In the current study, we demonstrated that metformin blocked neutrophils infiltration and decreased neutrophil‐derived MMP‐9 to inhibit TJ proteins degradation, prevent BSCB permeability, and eventually protect the spinal cord from secondary injury to improve long‐term functional recovery. And these effects were, at least in part, mediated *via* an AMPK‐dependent pathway. We also found that ICAM‐1 might be involved in metformin‐induced down‐regulation of neutrophil infiltration. Thus, our study provided evidence which suggested that metformin could have protective effects on SCI‐induced BSCB disruption, to improve neurobehavioral outcome.

Metformin, as a hypoglycaemic agent, is widely used for the treatment of Type 2 diabetes mellitus. The UK Prospective Diabetes Study (UKPDS) has reported that metformin administration clinically reduces the risk of all‐cause mortality and stroke, and exerts neuroprotective effects; however, these therapeutic effects are independent of its hypoglycaemic function. In after stroke period, metformin prevents neurons from nitrative stress and restored angiogenic signalling in the brain 39. In addition, it has been demonstrated that metformin attenuates the neurological deficits in ischaemic cerebral injury 40, 41. And according to our results, metformin could enhance functional recovery in rat SCI model. This study confirmed that metformin might provide therapeutic interventions for traumatic SCI.

It is well known that keeping integrity of BSCB could protect CNS stability to ameliorate SCI, and TJ proteins are essential components of the barrier 10. Thus, regulating TJ proteins may be involved in the metformin‐induced neuroprotection. However, studies regarding the effects of metformin on TJ proteins are controversial. Liu and *et al*. found that in middle cerebral artery occlusion mice model, metformin up‐regulates the level of TJ proteins to attenuate BBB disruption and to decrease infarct volume 23; however, *in vitro* studies from Takata and Xiang showed that metformin could not increase the expression of TJ proteins in brain capillary endothelial cells or submandibular gland cells 42, 43. Our results were consistent with Liu's work, our *in vivo* study demonstrated that metformin up‐regulated TJ proteins. At the meantime, our *in vitro* study revealed that there was no significant difference between vehicle control and metformin treatment in the level of TJ proteins of endothelial cells, which were consistent with Takata and Xiang's reports. These controversial results indicated that the function of preventing BSCB disruption by metformin might not be mediated *via* promoting the expression of TJ proteins, but *via* inhibiting the degradation of TJ proteins.

Although many factors are known to contribute to BSCB breakdown, MMPs plays a critical role in the barrier dysfunction in several pathological conditions 44, 45, 46. And MMP‐9 is among the most important MMPs in TJ proteins degradation 10, 12. Lee's study found that blocking MMP‐9 secretion by fluoxetine could alleviate TJ molecular degradation and BSCB disruption 10. It is also reported that 17β‐estradiol attenuates BSCB disruption to promote functional recovery by down‐regulating MMP‐9 13. Noble *et al*. found that MMP‐9‐null mice exhibited significantly less BSCB dysfunction, alleviation of neutrophil infiltration and significant locomotor recovery compared with wild‐type mice in SCI 12. Therefore, MMP‐9 is considered to be a therapeutic target for SCI‐induced BSCB breakdown.

Recent reports in tumours suggested that metformin could inhibit cell migration and invasion by suppression of MMP‐9 47, 48, 49. A study in skin tissue showed that metformin significantly inhibits MMP‐9 expression and protect collagen degradation from solar ultra‐violet radiation 50. It is also reported that metformin could reduce vascular remodelling and severity of haemorrhagic transformation by inhibiting MMP‐9 activity 51. Therefore, we investigated the level of MMP‐9 after metformin administration in SCI rats, our data demonstrated that metformin could down‐regulate MMP‐9 significantly, which contributed to decrease degradation of TJ proteins following SCI.

It is widely accepted that in CNS disease, the increased MMP‐9 primarily come from infiltrating neutrophils 15, 16, 17. Removing neutrophils from the circulation prior to SCI with an anti‐neutrophil serum significantly reduces MMP‐9 level, which indicates that the increase in MMP‐9 after SCI come from neutrophils 17. And emerging evidence shows that metformin is beneficial to block neutrophils infiltration in numerous pathological conditions. Bergheim and colleagues reported that the increased number of neutrophils in liver is significantly decreased by metformin treatment 52. In a myocardial infarction study, metformin is found to attenuate neutrophils recruitment in rats 53. In the present study, we investigated the effect of metformin on blocking neutrophils infiltration after SCI. Consistent with these previous studies, our results suggested that SCI‐induced neutrophils infiltration was attenuated by metformin administration, which also contributed to down‐regulate MMP‐9. These results showed the correlation between neutrophils inhibition and MMP‐9 down‐regulation. However, it is unclear that the decrease in MMP‐9 is due to inhibited production of MMP‐9 directly from neutrophils. Thus, we investigated the role of metformin on isolated neutrophils *in vitro*, in which no other cells existed to generate MMP‐9. As our results showed, metformin could down‐regulate TNF‐α‐induced MMP‐9 production in neutrophils. Together, these results indicated that metformin could directly inhibit the level of MMP‐9 in neutrophils.

Metformin could attenuate neutrophils recruitment in SCI, but the potential mechanisms remain to be illustrated. ICAM‐1 is a adhesion molecule, which is expressed in many types of cells especially endothelial cells 54. ICAM‐1 is expressed constitutively in endothelial cells at low level, and its expression is significantly increased after tissue injury 55. It is well known that ICAM‐1 expresses in endothelial cells to facilitate neutrophils infiltration, and regulating adhesion molecules are found to affect the infiltrating neutrophils and integrity of BBB in CNS injury 23, 56, 57. Therefore, we hypothesized, whether metformin‐induced resistance of neutrophilic exudate was associated with ICAM‐1 down‐regulation. And our results showed that expression of ICAM‐1 was significantly down‐regulated by metformin in SCI rat, which indicated that ICAM‐1 might be involved in metformin‐induced resistance against neutrophil infiltration. Future studies are expected to elucidate‐specific mechanisms.

However, depleting neutrophils by anti‐Ly6G antibody after SCI is found to worsen the neurological outcome 58. This report is contradicts with previous studies as well as our results, which showed that limiting neutrophil infiltration by certain drugs could promote SCI recovery 13, 59. The potential reasons might be as follow: the number of neutrophils could be depleted to very low level using an anti‐Ly6G antibody, but relevant drugs decreased neutrophils to a degree of relatively moderate level; using an anti‐Ly6G antibody could decrease the systemic neutrophils, but relevant medicine could only decrease neutrophils in the site of injury. It should be also noted that neutrophils could release vascular endothelial growth factor to promote wound repair, which might be helpful to SCI 60. Thus, further studies are expected to clarify this issue.

We demonstrated that metformin could inhibit MMP‐9 in neutrophils, but to understand potential mechanisms in SCI, we did further investigations. Other studies indicated that the pleiotropic effects of metformin are mediated *via* activation of AMPK 61, 62. And it is also found that activating AMPK significantly suppress MMP‐9 activity to promote cell/extracellular matrix stability 63, 64. In the current study, we detected AMPK signalling pathway in neutrophils *in vitro*. And we demonstrated that metformin promoted phosphorylation of AMPK in neutrophils, and inactivating AMPK by compound C abolished the metformin‐induced MMP‐9 down‐regulation. These results suggested that the effect of metformin on the MMP‐9 production was AMPK‐dependent. But the underlying mechanisms of AMPK effect on MMP‐9 are still unknown. It has been reported that the p65 subunit of NF‑κB binding to the promoter of the MMP‐9 gene could be suppressed by AMPK activation 65. In addition, it has been found that chitosan oligosaccharides treatment increased AMPK activity, suppressed the NF‐κB‐mediated MMP‐9 production 66. Therefore, it is believed that NF‐κB might be related to AMPK‐induced MMP‐9 decrease. Thus, more effort is needed to be done.

All of these results indicate that metformin‐conferred neuroprotection might be mediated *via* BSCB protection, which involved (at least in part) inhibition of neutrophil infiltration and suppression of MMP‐9 in neutrophils by AMPK activation. In further studies, MMP‐9‐deficient or neutrophil‐depleted mice can be used to provide more direct evidence for mechanisms of metformin‐induced BSCB protection. Compound C might also activate potential non‐specific effects. Thus, isolated neutrophils from AMPK‐deficient mice could be studied to investigate whether there were other underlying mechanisms. Although increased MMP‐9 primarily comes from infiltrating neutrophils after CNS injury, the effects of metformin on other multiple cell types should also be explored in the future 15, 16, 17.

In conclusion, we demonstrated that metformin ameliorated BSCB disruption through blocking neutrophils infiltration and decreasing the MMP‐9, to down‐regulate TJ proteins degradation. AMPK might be involved in MMP‐9 regulation of neutrophils. Our study is helpful to better understand the working mechanism of the therapeutic effect of metformin on SCI.

## Conflict of interest

The authors declare no conflict of interest.

## Supporting information


**Figure S1** Metformin prevented the loss of TJ proteins after SCI. (a, b) Representative western blots and quantification data of Occludin, Claudin‐5, ZO‐1 and β‐actin at 5 days and 7 days after SCI, # represents *P* < 0.05 versus the SCI group, *n* = 5.
