# Supplementary figures and images for: Metformin ameliorates BSCB disruption by inhibiting neutrophil infiltration and MMP‐9 expression but not direct TJ proteins expression regulation
Source: J Cell Mol Med. 2017 Jul 12;21(12):3322–36. doi: 10.1111/jcmm.13235 (PMC5706495; doi:10.1111/jcmm.13235)

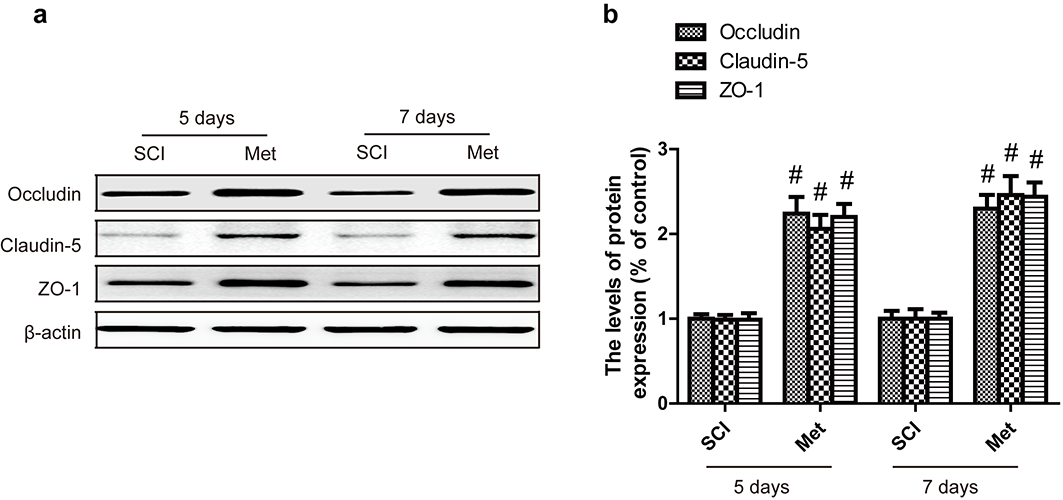

Supplement: Supplementary file 1 — Figure S1 Metformin prevented the loss of TJ proteins after SCI. (a, b) Representative western blots and quantification data of Occludin, Claudin‐5, ZO‐1 and β‐actin at 5 days and 7 days after SCI, # represents P < 0.05 versus the SCI group, n = 5. [file JCMM-21-3322-s001.tif]
